# Cerebrospinal fluid leakage following penetrating spinal trauma in a patient with congenital spinal dysraphism: A case report

**DOI:** 10.1016/j.ijscr.2025.111123

**Published:** 2025-03-04

**Authors:** Behnaz Rahatijafarabad, Hooman Koohestani

**Affiliations:** Department of Neurosurgery, Golestan University of Medical Science, Golestan Province, Iran

**Keywords:** Cerebrospinal fluid leak, Trauma, Spine, Spinal dysraphism

## Abstract

**Introduction:**

Spinal dysraphism, a congenital condition characterized by vertebral malformations like spina bifida occulta, may lead to cerebrospinal fluid (CSF) leaks following a minor trauma. We report a case of a 19-year-old male with congenital spinal dysraphism who developed a CSF leak after a stab wound to the lumbosacral region.

**Presentation of case:**

The patient was admitted four days post-injury with CSF leakage, vertigo, and nausea but no neurological deficits. Imaging revealed a dural tear and spina bifida occulta. The combined surgical intervention, including primary closure, dural repair, and medical intervention, was remarkably successful, significantly improving the patient's condition.

**Discussion:**

This case underscores the urgent need for early detection and treatment of CSF leaks in patients with congenital spinal dysraphism who experience trauma. Spinal dysraphism can significantly increase the risk of complications following traumatic injuries. The absence of immediate symptoms and underlying anomalies complicates diagnosis and treatment, emphasizing the need for thorough evaluation using imaging studies to identify potential dural defects after spinal trauma.

**Conclusion:**

This case highlights the critical relationship between congenital spinal dysraphism, primarily spina bifida occulta, and spinal traumatic injuries. It also highlights the importance of accurate diagnosis and early intervention to improve patient outcomes and prevent complications.

## Highlights

• Spinal dysraphism is a congenital condition characterized by incomplete spinal fusion, which can lead to spina bifida occulta.
• Patients with spinal dysraphism face a higher risk of CSF leaks after trauma, even without neurological deficits.
• CSF leaks may present with vertigo and nausea, requiring prompt recognition to avoid diagnostic delays.
• Imaging studies, mainly CT and MRI, are essential for detecting dural defects in traumatic spinal injuries.
• Timely surgery, including dural repair and lumbar drainage, helps restore CSF flow and prevent complications.

## 1 Introduction

The clear, colorless liquid that surrounds and fills the subarachnoid space (SAS) is called cerebrospinal fluid (CSF). It is mostly water (99 %), with the remaining 1 % of proteins, carbohydrates, electrolytes, and neurotransmitters. A cerebrospinal fluid leak is a leak of the fluid surrounding the brain and spinal cord, and it happens when there is a rupture or break in the dura mater [1,2]. Symptoms include orthostatic headache, nausea, and neck discomfort, with less common symptoms such as tinnitus and loss of taste or smell [2,3]. CSF leaks are most commonly traumatic (80 %), followed by iatrogenic (16 %), and spontaneous (4 %) [2].

Spinal dysraphism is an incomplete spine fusion during embryogenic phases and includes different congenital abnormalities categorized as open and closed [4]. Closed spinal dysraphism, known as Spina Bifida Oculta, makes its diagnosis challenging as the neural tissue is covered with skin [5]. The incidence of spina bifida occulta is 10 % in adults [6]. Although CSF leaks from traumatic spinal injury are well documented, their co-occurrence with congenital spinal dysraphism, particularly spina bifida occulta, is uncommon [7,8].

This report highlights the rare condition of a young adult with spinal CSF leakage following a stab wound injury in the lumbosacral region without any neurological deficit. This patient has congenital spinal dysraphism without any prior medical history, which was managed by surgery and medical interventions. The work has been reported in line with the SCARE criteria [9].

## 2 Case presentation

A 19-year-old man was admitted to the emergency room four days after a stab wound in his lumbosacral region, presenting with colorless fluid leakage from the wound. Four days earlier, he had a penetrating trauma in his lumbosacral region from behind with a stab wound, which had been sutured without any complication at a primary local hospital. In the first two days after the injury, he did not notice any leakage; on the third day after bandage removal, he noticed a fluid leakage from his suture site and approached the same local hospital, which was referred to our hospital. He also complained of vertigo upon standing and nausea since two days ago. Any other symptoms, were not detected. No prior significant medical history was reported. The skin wound was 3 cm at the midline of lower lumbar region. The patient was stable and showed no infection, or inflammation around the wound. The cranial and peripheral nerve examination was normal, and upper and lower limbs had full force without any local neurological deficit. The patient was alert and without fever; Kernig's and Brudzinski's signs were negative.

**Fig. 1:**
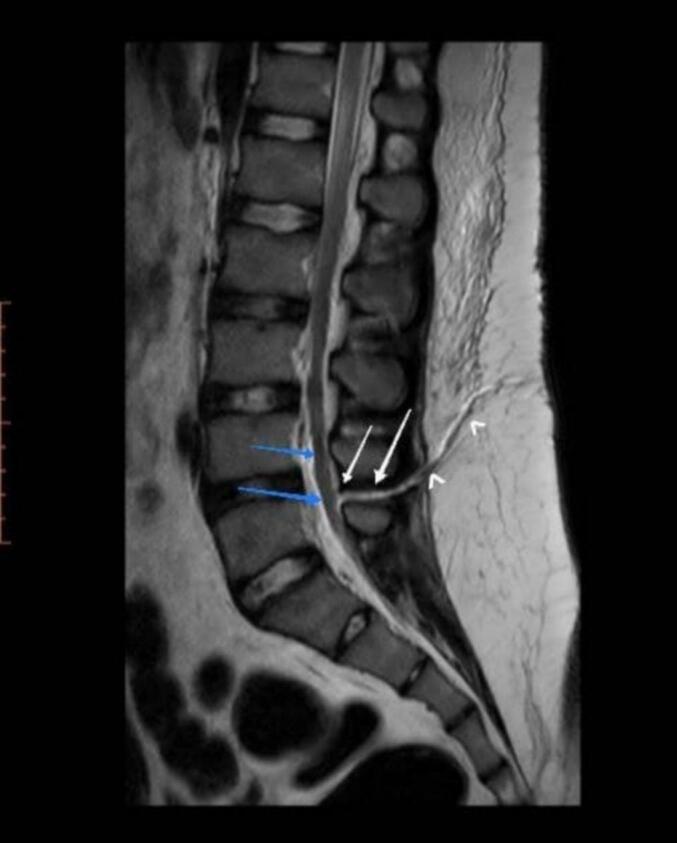
Axial CT scan of the lower lumbar region, white arrows show penetrating trauma at interspinous L4-L5 space, and the fistula tract extends from subcutaneous up to epidural space of spinal canal at L4-L5 level. Blue arrows show nerve roots clumping. (For interpretation of the references to colour in this figure legend, the reader is referred to the web version of this article.)

A lumbosacral Computed tomography (CT) scan without contrast and Magnetic resonance imaging (MRI) was requested, and empiric antibiotic therapy was started with a complete bed rest order. The initial imaging showed a localized dural tear with evidence of CSF leakage through a fistula from epidural space up to subcutaneous at the L4-L5 level (Fig. 1).

**Fig. 2:**
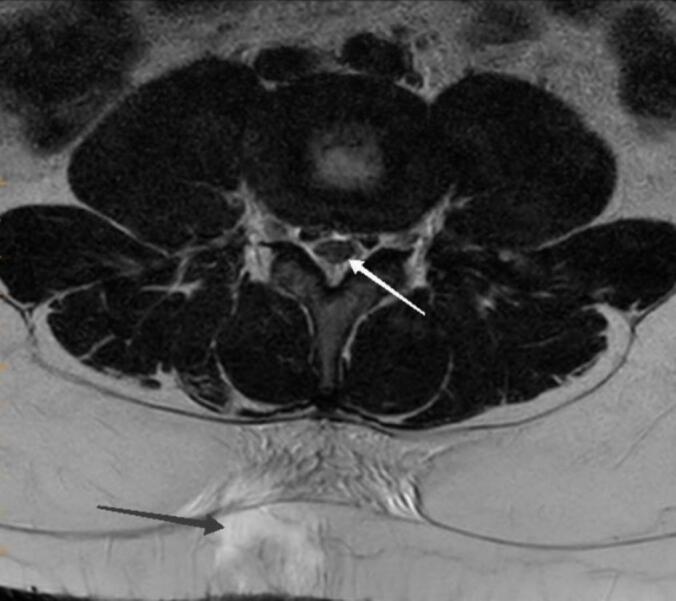
Axial T2-weighted lumbar MRI image, white arrow show distortion and clumping of nerve roots at the level of injury, according to spinal bifida occulta in lumbosacral levels. Black arrow show CSF effusion.

A bone defect was observed in the right side lamina of the fourth lumbar spine. Also, the MRI revealed abnormal thickening of the epidural space with high T1 and T2 spinal intensity at the lower lumbar level and distortion and clumping of nerve roots. According to Spina bifida occulta at the sacral level, the finding suggested epidural lipomatosis (Fig. 2).

On the second day of admission, surgery was indicated for the patient, including a primary closure with partial laminectomy, dural repair and augmentation by fat and muscle flap, and a lumbar drain insertion at the subarachnoid space for five days. The patient responded well to the medical management, which included a Carbonic Anhydrase Inhibitor (Acetazolamide), a wide-spectrum antibiotic therapy, and Trendelenburg positioning. Two days after surgery, a Lumbar Puncture was performed. The results showed CSF glucose (71 mg/dl) and protein (78.4 mg/dl); WBC was not seen.

The patient was discharged from the hospital ten days after surgery. A follow-up examination a month later showed persistence of symptom resolution and a well-healed surgical site. Due to the complete resolution of the symptoms and signs and the patient's financial limitations, no further imaging was ordered. The patient is now following a routine checkup plan every three months, ensuring ongoing care and monitoring.

## 3 Discussion

### 3.1 Pathophysiology and presentation

**Fig. 3:**
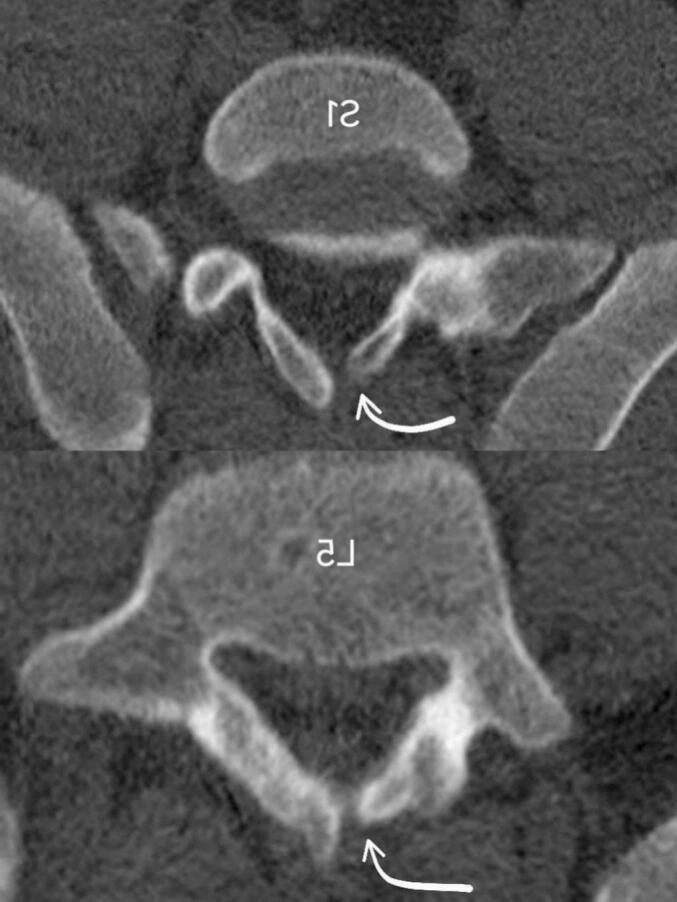
Axial CT scan of lumbosacral region, white arrows show a bone defect in L5 and S1 lamina with sclerotic margins.

CSF leak is a rare complication in spinal trauma patients, affecting approximately 5 out of 100,000 cases annually in the US [10]. The most common fracture patterns associated with traumatic epidural tears are lumbar burst fractures with associated vertical lamina fractures, where the dura may become trapped [1]. In our case, the patient presented with a localized defect in the L5 lamina, which has no neurological deficit, as shown in Fig. 3.

Patients with spontaneous spinal CSF leaks typically appear with a positional headache. Additional typical symptoms include nausea, photophobia, hyperacusis, and stiff neck [11]. In the Luszczyk et al. study, about 83 % of patients who experienced a traumatic dural rupture as a result of a spine injury had a neurologic deficit [1]. Another study demonstrated that 2.7 % to 4.5 % of patients with spinal cord stabbing injuries did not exhibit any neurological symptoms or signs [10].

### 3.2 Differential diagnosis and diagnostic approach

Considering the clinical presentation, the first potential diagnosis was a dural tear due to a laminar fracture, which was ruled out after diagnosis of underlying spina bifida oculta in this patient. Liu et al. study suggests spina bifida oculta as a predisposing factor for spinal injury. Chronic traction of the spinal cord could be a risk factor for spinal injury following a minor trauma [12]. In this case, the presence of spina bifida oculta despite no apparent abnormality and its underlying bone defect made the patient more vulnerable to dural damage as a result of a traumatic spinal injury.

Rocha Filho et al., in a similar case study, mention in cases where low-energy trauma leads to a minor sacral fracture and subsequent CSF leak; a pre-existing anatomical abnormality may be involved. Conditions like meningeal cysts and spina bifida occulta can increase the risk of sacral bone remodeling and weakness [13]. While CSF leaks are commonly associated with trauma, the occurrence of spina bifida occulta alongside such a leak after a penetrating spinal injury is rare. Cerebrospinal fluid leaks can be localized and characterized by imaging modalities like MRI or CT [10,11].

### 3.3 Management strategy

As far as we can search in the literature, our case was the only case of the CSF leak following a penetrating trauma complicated with spinal Bifida occulta. Due to the underlying anomaly and persistence of the CSF leak for four days, a combination of conservative and surgical approaches was applied. Most CSF leaks following a minor trauma would be treated by conservative treatment. Conservative treatment usually involves bed rest, wound care, and infection prevention [13].

Ramirez-Ferrer et al., in their case report on conservative management of a CSF leak in penetrating spinal trauma, suggests Acetazolamide effectively closes the primary defect in spontaneous CSF leaks [10,14]. We conducted a similar conservative management for our patient by ordering Acetazolamide, a wide-spectrum antibiotic therapy, hydration, and Trendelenburg positioning. Late diagnosis of the CSF leak in this patient could lead to complications like meningitis, which was managed by administering prophylactic broad-spectrum antibiotics and close monitoring by post-surgery lumbar puncture.

Numerous surgical techniques are available to treat a CSFL, including fibrin glue, direct replacement of collagen patches, blood-soaked gelatin sponge, and direct primary dura closure. The methods have not been shown to differ significantly from one another. As the dural tear may not be identified in most cases, closure with a muscle flap plus fibrin-based sealants may be required [10,14]. Banno et al. in 2012 reported a case of sacral pseudomeningocele with spina bifida Occulta following a car accident and blunt trauma, which was managed by a free fat graft with fibrin glue due to tight adherence of dural edge to vertebral lamina [6]. In our case, due to a dural defect, a dural repair and augmentation by fat and muscle flap was performed. Also, we used an intradural continuous drainage catheter to decrease intradural pressure and avoid fluid outflow through the CSFL for five days. Usually, the lumbar drain treatment aims to provide short-term symptom relief while allowing the CSF leak to heal [2].

The main limitation of this report is the lack of similar documented cases or management protocols, limiting evidence-based conclusions. Restricted access to diagnostic tools (CT/MRI) in local hospitals delayed diagnosis, highlighting the need for improved local diagnostic capacities and an enhanced referral system. Additionally, the absence of pre-injury imaging challenges the assessment of pre-existing anomalies, emphasizing the importance of baseline imaging in high-risk individuals.

## 4 Conclusion

In conclusion, this case underscores the critical role of congenital spinal dysraphism in traumatic spinal dural tears, underscoring the need for timely diagnosis and imaging to uncover underlying conditions. Vigilant monitoring for complications such as meningitis is crucial for patients with delayed approach, ensuring timely intervention. Moreover, implementing enhanced imaging protocols and rigorous follow-up plans can enhance outcomes by facilitating the early detection and management of spinal CSF leaks and associated injuries.

## Author contribution

• Conception or design of the work - Hooman Koohestani
• Data collection - Behnaz Rahatijafarabad
• Data analysis and interpretation - Behnaz Rahatijafarabad
• Drafting the article - Behnaz Rahatijafarabad
• Critical revision of the article - Hooman Koohestani
• Surgeon and study supervision - Hooman Koohestani

## Consent

Written informed consent was obtained from the patient for publication of this case report and accompanying images. A copy of the written consent is available for review by the Editor-in-Chief of this journal on request

## Ethical approval

The Golestan University of Medical Science Ethics Committee's has approved this case report. Approval ID: IR.GOUMS.REC.1403.247.

## Guarantor

Behnaz Rahatijafarabad.

## Research registration number

This case report doesn't include any ‘First in Man’ procedure.

## Funding

The authors of this study declare that there are no funding sources.

## Conflict of interest statement

There are no conflicts of interest to declare.

## References

[1] Luszczyk M.J., Blaisdell G.Y., Wiater B.P., Bellabarba C., Chapman J.R., Agel J.A. (2014). Traumatic dural tears: what do we know and are they a problem?. Spine J..

[2] Severson M., Schaurich C.G., Strecker-McGraw M.K. (2023). StatPearls [Internet].

[3] Rajpal S., Nambiar M., Castanelli D., Khabaza A., Asadi H., Jhamb A. (2023). Spontaneous intracranial hypotension and spinal epidural CSF leaks: diagnosis and management. J. Clin. Neurosci..

[4] Chellathurai A., Kathirvelu G., Mukkada P.J., Rajendran K., Ramani R. (2021). Spinal dysraphisms: a new anatomical–clinicoradiological classification. Indian J. Radiol. Imaging.

[5] Holmes L.C., Li V. (2019). Occult spinal dysraphism. Pediatr. Rev..

[6] Banno T., Ohishi T., Suzuki D., Honda Y., Kobayashi S., Matsuyama Y. (2012). Traumatic sacral pseudomeningocele with spina bifida occulta: case report. J. Neurosurg. Spine.

[7] Badejo O.A., Shokunbi M.T., Adeolu A.A., Oderinde I.O., Akinmoladun J.A., Ogbole G.I. (2025). Atypical variants of spinal dysraphism: a case series. J. West Afr. Coll. Surg..

[8] Pillai S.S. (2022). Spinal dysraphism. Kerala J. Orthopaed..

[9] Sohrabi C., Mathew G., Maria N., Kerwan A., Franchi T., Agha R.A. (2023). The SCARE 2023 guideline: updating consensus Surgical CAse REport (SCARE) guidelines. Int. J. Surg..

[10] Ramirez-Ferrer E., Abaunza-Camacho J.F., Pineda-Martinez A.F., Aguilera-Pena M.P., Riveros-Castillo W.M., Laverde-Frade L. (2022). Cerebrospinal fluid leak following penetrating trauma to the spine without neurological deficit: a case report. Surg. Neurol. Int..

[11] Jones M.R., Shlobin N.A., Dahdaleh N.S. (2021). Spontaneous spinal cerebrospinal fluid leak: review and management algorithm. World Neurosurg..

[12] Liu G., Jiang W., Tang X., Tan S., Zhang M., Tao L. (2022). Spina bifida occulta is a risk factor for spinal cord injury without fracture or dislocation for children performing a backbend during dance. Front. Pediatr..

[13] da Rocha Filho M.R.M., Dwan V.S.Y., Edelmuth D.G.L., Helito P.V.P., Amaral D.T., de Paula Correa M.F. (2024). Unsuspected cerebrospinal fluid leak following a minor sacral fracture: a case report. Radiol. Case Rep..

[14] Woodroffe R.W., Nourski K.V., Helland L.C., Walsh B., Noeller J., Kerezoudis P. (2018). Management of iatrogenic spinal cerebrospinal fluid leaks: a cohort of 124 patients. Clin. Neurol. Neurosurg..

